# Dietary interventions in autism: a critical appraisal and commentary on the findings of a systematic review

**DOI:** 10.12968/bjnn.2024.0035

**Published:** 2024-08-02

**Authors:** S Woods, A Doherty, J Hill

**Affiliations:** Lancashire and South Cumbria NHS Foundation Trust; University of Central Lancashire

## Abstract

An estimated 1% of the global population is believed to be autistic. Clinical focus is often on interventions that target social functioning, sensory processing and communication. Dietary interventions are often explored as a means of targeting these core symptoms. However, research findings are often inconclusive due to small sample sizes. This commentary critically examines a meta-analysis focused on dietary interventions - including omega-3, vitamins, and other supplements - in the treatment of autism. It evaluates the study’s findings and contextualizes their implications for neurological nursing practice.

## Introduction

Autism is a neurodevelopmental difference; where the brain works differently than those of neuro-typical peers, in areas such as social communication, sensory processing and interactions with the environment (i.e. the presence of restricted and repetitive behaviours, interests and/or activities) (American Psychiatric Association, 2013). It is estimated that 1% of the population is autistic (World Health Organisation, 2023). However, due to intersectionality disparities within the clinical diagnosis research, including gender and race, it is likely that this prevalence figure may be higher (Brickhill et al., 2023). The underlying causative mechanisms are not well understood, although it is believed that there is a significant genetic, biological and environmental interplay (Rutter, 2005; Sandin et al., 2014).

As there is no cure for autism, a significant proportion of the research has focussed on speech and behavioural interventions with the view of improving autistic individual’s social functioning, sensory processing and communication (Brignell et al., 2018). These include educational, psychosocial and pharmacological interventions (Kalra, Gupta, & Sharma, 2023). An area that has received increasing attention is that of dietary intervention, including specific diets and supplementation (Amadi et al., 2022). Autistic individuals, specifically autistic children, often show a high level of food selectivity and a strong aversion to trying unfamiliar foods (Esposito et al., 2023). It is therefore not uncommon for gastrointestinal (GI) issues, such as constipation, diarrhoea and/or abdominal pain, to be regularly reported by the autistic population (Gan et al., 2023) with the suggestion that the limited nutritional quality and composition also alters the microbial composition of the gut (Sivamaruthi et al., 2020). Consequently, the intake and absorption of vitamins, minerals, and essential fatty acids are therefore inadequate (Esposito et al., 2023). As a result, targeted dietary interventions are utilised with the view to not only improve nutritional status, but also behavioural changes generated by nutritional deficiency (Barnhill et al., 2016).

A wide range of dietary interventions have been explored looking at both restrictive and supplementary methods (Sathe et al., 2017). Restrictive methods such as gluten-free and caseinfree (GFCF) diets have shown possible benefits within the literature (Knivsberg et al., 2002; Quan et al., 2022). However, there have been questions regarding the relatively small sample sizes used within the studies and the possible methodological concerns (Monteiro et al., 2020; Quan et al., 2022). There are also nutritional concerns regarding implementing further dietary restriction on a limited diet already (Jordan, 2018). Other areas of exploration are that of supplementation; specifically, that of omega-3 and specific vitamins and minerals (Pancheva et al., 2024). Although a favoured approach by parents, research has found no robust evidence to support its clinical use in the management of autistic core symptoms (Monteiro et al., 2020). As nurses are often approached regarding nutrition advice, it is important for them to keep up to date with the current evidence base regarding this condition (Eaton et al., 2022; Murphy & Girot, 2013).

### Aim of commentary

This commentary aims to critically appraise the methods used within the review Fraguas et al. (2019) and expand upon the findings in the context of clinical nursing practice (Fraguas et al., 2019).

### Critical appraisal of the methods used by Fraguas et al. (2019)

Using the AMSTAR 2 critical appraisal tool for systematic reviews (Shea et al., 2017), this meta-analysis satisfied 8 out of 16 criteria (see Table 1 for full AMSTAR 2 critical appraisal). Despite this relatively low criteria achievement, the main areas of concern were not deemed to be severe methodological issues. The main areas of concerns were the absence of a published protocol prior to the review’s commencement. Even though protocol registration of a systematic review is not always undertaken (van der Braak et al., 2022) it is important in reducing the possibility of reporting bias, enhancing transparency of the methods used in the systematic review, reducing potential duplication of research by others, and it assists in terms of reproducibility and reliability (Higgins et al., 2011; Pieper & Rombey, 2022). Another area of concern was the lack of independence in the screening and data extraction process. Evidence suggests that the gold standard should be dual screening independently (Stoll et al., 2019; Waffenschmidt et al., 2019). Although both screening and data extraction was carried out by two authors – it was not done so independently. This may have resulted in a possible increase in studies being overlooked for inclusion in the review, and possible errors in data extraction. The final area of concern was regarding the review’s restriction to include English language studies only. However, previous assessment of this potential methodological issue has demonstrated limited impact regarding the overall conclusion of a systematic review (Dobrescu et al., 2021). The remaining areas of concern were regarding (i) justification of Randomised Controlled Trial (RCT) study type only; (ii) no list of excluded studies and reasons for exclusion; and (iii) no lists of the funding sources for included studies (although the role of the funding source was accounted for within the quality assessment).

### Main findings of Fraguas et al. (2019)

After eliminating duplicates, 2,283 studies were identified, of which 27 double-blind RCTs were incorporated into the systematic review. The included RCTs scored between two and six out of a possible quality assessment score of six, with the majority of studies scoring five and six. Using a Meta-Regression the quality score did not seem to be an important moderating factor for effect size. Similarly, when able to be assessed, there was no evidence of association for the moderating factors including: year of publication, length of intervention, sample size, mean age and percentage of females.

When meta-analysed a statistically significant (p = >0.05) small effect (standard means difference [SMD] = <0.5) was found when comparing dietary supplementation (combination of omega-3 and vitamin supplementation and others) on anxiety, autistic general psychopathology, behavioural problems and impulsivity, global severity, hyperactivity and irritability, general language, social-autistic and stereotypies and restricted and repetitive behaviours, associated symptoms, autism global, clinical global impression and core symptoms compared to placebo. There was no evidence of difference (p = >0.05) for the outcomes of cognition, sensory and motor, and sleep.

When omega-3supplementation was used there was a statistically significant small effect observed for the outcomes of social autistic and stereotypies, general language, associated symptoms and core symptoms compared to placebo. There was no evidence of difference for the outcomes of autistic general psychopathology, global severity cognition, cognition, hyperactivity and irritability, stereotypies and restricted and repetitive behaviours, autism global and clinical global impression.

When using vitamin supplementation alone there was a statistically significant small effect for the outcomes of behavioural problems & impulsivity, global severity, hyperactivity and irritability, language, stereotypies, restricted & repetitive behaviours, associated symptoms, clinical global impression and core symptoms compared to placebo. There was no evidence of difference for autistic general psychopathology, social-autistic and autism global.

For the subgroup analysis for children and adolescents there was no evidence of difference for supplementation (omega-3, vitamin supplementation, and/or other supplementation), omega-3 supplementation and vitamins for the outcomes of language, associated symptoms, autism global, clinical global impression and core symptoms compared to the main analysis. For geographical location of the study: there was no evidence of differences between USA, Europe and other countries for supplementation (omega-3, vitamin supplementation, and/or other supplementation) for the outcomes of social-autistic, stereotypies, restricted and repetitive behaviours, core symptoms and associated symptoms. Although, the European studies found a statistically significant small effect in language.

### Commentary

The findings of this meta-analysis suggest potential benefits of global dietary supplementation, omega-3 and vitamin supplementation in alleviating certain core and associated symptoms of autism. More recent studies, particularly those focused on children, have supported the use of various dietary supplements (Doaei et al., 2021; Javadfar et al., 2020), contrasting with findings of limited effect in some other studies (de Andrade Wobido et al., 2022; Siafis et al., 2022). Notably, the effects observed in this meta-analysis were modest (small effect). The authors noted methodological variation amongst the included studies, encompassing variations in intervention type, clinical outcome measures, and sample characteristics, thereby limiting the external validity of their findings. Although, when tested, these characteristics and quality of studies did not appear to make a significant difference to the small effects observed within the range of certain core and associated symptoms of autism. Consequently, these findings do not alter recommendations at the national or international level regarding the use of specific vitamins, minerals, and dietary supplements in the management of autism (Hyman, Levy, & Myers, 2020; National Institute for Health and Care Excellence, 2012). Both guidelines indicate that due to inconclusive evidence, use of vitamins, minerals or dietary supplements in the management of core features of autism are not recommended.

The authors observed a small effect between omega-3 supplementation and outcomes of social autistic and stereotypies, general language, associated symptoms and core symptoms compared to placebo. However, a more recent review by Jiang at al. (2023) found that effects of omega-3 supplementation on autism were too weak to conclude that core symptoms were alleviated (Jiang et al., 2023). This aligns with the evidence underpinning general use of omega-3 supplementation (often explored in coronary health) (Abdelhamid et al., 2020). However this does not negate the potential benefits of including omega-3 rich foods in the diet (Iso et al., 2006).

Although the authors also aimed to examine restrictive diets as an intervention for autism, the selected data was unfeasible for inclusion due to a lack of studies assessing consistent predictor variables and clinical outcomes. Consequently, no general dietary recommendations were supported by the authors. Indeed, in previous review by Lange et al. (2015), it was concluded that most investigations assessing the efficacy of restrictive diets, such as the GFCF diet, were flawed, and the evidence to support the therapeutic value of these diets was limited and weak (Lange, Hauser, & Reissmann, 2015). Indeed Quan et al. (2022) showed that a GFCF diet can reduce stereotypical behaviours and improve the cognition of children with autism (Quan et al., 2022). However, there was large heterogeneity within the studies and small sample sizes. Current national guidelines do not advocate the use of exclusion diets in the management of autism (National Institute for Health and Care Excellence, 2012).

Dietary approaches are frequently explored as potential treatments for autistic individuals, yet the evidence base remains flawed and inconsistent. This inconsistency is not surprising, given the individual variability within the autistic population, particularly when viewed through the lens of neurodiversity (Palmer et al., 2015). Neurodiversity emphasizes viewing the autistic brain as natural, albeit different from societal norms, rather than defective (Jaarsma & Welin, 2012). Moreover, the lack of evaluation of nutritional and physical status at baseline may contribute to diagnostic overshadowing, potentially affecting the reliability of outcome measures. For instance, the autism research charity *Autistica* has advocated for the adoption of strengths-based approaches to autism in research and clinical practice (Huntley, 2019). Future research in this field should encompass a multifaceted approach. In addition to incorporating baseline nutritional data to prevent diagnostic overshadowing, research should prioritize understanding the lived experiences of autistic individuals and their families. This involves seeking their views and perspectives on navigating a neurotypical world, and identifying strategies that they find useful and empowering. Furthermore, exploring the impact of environmental adjustments and adaptations in managing core and associated symptoms of autism could offer valuable insights into optimizing support and intervention strategies. Although dietary interventions (exclusion diets/supplementation) are not advocated in the management of autism, dietary strategies should be considered on an individual basis (ideally by a registered dietitian) when there are nutritional deficiencies, intolerances or allergies (National Institute for Health and Care Excellence, 2011, 2012).

### CPD reflective questions

What are the main limitations of this review and meta-analysis?

What advice would you provide on the use of supplements (including omega-3, vitamins and/or other supplements) in the management of autism symptoms?

What other factors and/or adaptions should be considered when working with autistic people?

## Figures and Tables

**Table 1:** Critical appraisal using the AMSTAR-2 tool for assessing systematic reviews

AMSTAR 2 items	Responses
1. Did the research questions and inclusion criteria for the review include the components of PICO?	Yes - Only randomised controlled trials [RCTs] in English which compared ‘autism spectrum disorder (ASD)’ and dietary intervention were included.
2. Did the report of the review contain an explicit statement that the review methods were established prior to the conduct of the review and did the report justify any significant deviations from the protocol?	No- no written protocol prior to conduct of review/no protocol registered.
3. Did the review authors explain their selection of the study designs for inclusion in the review?	No- There was no explanation of why only RCTs only were included.
4. Did the review authors use a comprehensive literature search strategy?	No - This review carried out a 2-step literature search; a comprehensive multi-database search and a manual search of the references lists of the articles included in the meta-analysis for any studies not identified in the multi-database literature search. The search occurred from database inception to September 2017. However, authors did not justify publication restrictions such as including English Language publications only.
5. Did the review authors perform the study selection in duplicate?	No - Three reviewers, though not independently, completed the screening of the abstract title and full-text paper. Arbitration was resolved through discussion and consensus.
6. Did the review authors perform data extraction in duplicate?	No- Data was extracted by two reviewers and verified by a further two reviewers.
7. Did the review authors provide a list of excluded studies and justify the exclusions?	No- no list of excluded studies and justification for exclusions were made available
8. Did the review authors describe the included studies in adequate details?	Yes - a full list of all study characteristics presented.
9. Did the review authors use a satisfactory technique for assessing the risk of bias in the individual studies that were included in the review?	Yes- An overall assessment of evidence quality was conducted using an item checklist inspired by the Cochrane Collaboration’s tool (Higgins *et al.* 2011). However, there was no indication that this process was undertaken in duplicate or independently.
10. Did the review authors report on the sources of funding for the studies included in the review?	No - there is no indication of any funders regarding the included studies.
11. If meta-analysis was performed did the review authors use appropriate methods for statistical combination of results?	Yes- Evidence synthesis was undertaken using a random-effects model with heterogeneity being assessed through visual inspection of forest plots and the I^2^ statistic. A meta-analytic subgroup analysis included studies assessing only children and young people as well as by region (United States, Europe and other regions). A series of Meta-Regression analyses to examine potential moderating factors, such as study quality, year of publication, intervention duration, sample size, average age, and the percentage of female participants was performed.
12. If meta-analysis was performed did the review authors assess the potential impact of Risk of Bias (RoB) in individual studies on the results of the meta-analysis or other evidence synthesis?	Yes - this review carried out a subgroup analysis assessing the effects of the varying quality levels of included studies on the varying effect levels.
13. Did the review authors account for RoB in individual studies when interpreting/discussing the results of the review?	Yes - it was highlighted that there was no notable change in effect based on quality of included studies.
14. Did the review authors provide a satisfactory explanation for, and discussion of, any heterogeneity observed in the results of the review?	Yes - a range of subgroup analyses were undertaken to explore the various possible causes of heterogeneity.
15. If they performed quantitative synthesis did the review authors carry out an adequate investigation of publication bias (small study bias) and discuss its likely impact on the results of the review?	Yes - an Eggers test was undertaken for all comparisons even though in some cases they may have been underpowered due to limited number of studies
16. Did the review authors report any potential sources of conflict of interest, including any funding they received for conducting the review?	Yes - All conflicts of interests for all offers were acknowledged.

## Key Points

- Potentially, there are small benefits to be gained from global dietary supplementation, omega-3 and vitamin supplementation in alleviating certain core and associated symptoms of autism.
- However, further evidence is needed to ascertain the potential benefits of both omega-3 and vitamin supplementation, to inform the establishment of standard recommendations for individuals with autism.
- Future research is needed to identify the types of moderating factors that may influence the effectiveness of these dietary supplements for people with autism.
